# Do Higher Radiation Doses with Concurrent Chemotherapy in the Definitive Treatment of Esophageal Cancer Improve Outcomes? A Meta-Analysis and Systematic Review

**DOI:** 10.7150/jca.44447

**Published:** 2020-05-18

**Authors:** Linlin Xiao, Brian G. Czito, Qingsong Pang, Zhouguang Hui, Shaowu Jing, Baoen Shan, Jun Wang

**Affiliations:** 1 Department of Radiotherapy, Fourth Hospital of Hebei Medical University, Shijiazhuang, Hebei, China.; 2 Department of Radiation Oncology, Duke University, Durham, North Carolina, USA.; 3 Department of Radiotherapy, National Clinical Research Center for Cancer, Tianjin's Clinical Research Center for Cancer, Key Laboratory of Cancer Prevention and Therapy, Tianjin Medical University Cancer Institute and Hospital, Tianjin, China.; 4 Department of Radiotherapy, Cancer Institute & Hospital, Peking Union Medical College, & Chinese Academy of Medical Sciences, Beijing, China.

**Keywords:** definitive concurrent chemoradiotherapy, dose escalation, esophageal cancer, esophageal squamous cell carcinoma, radiation dose

## Abstract

**Background**: To investigate the effects and safety profile of radiation dose escalation utilizing computerized tomography (CT) based radiotherapy techniques (including 3-Dimensional conformal radiotherapy, intensity-modulated radiotherapy and proton therapy) in the definitive treatment of patients with esophageal carcinoma (EC) with definitive concurrent chemoradiotherapy (dCCRT).

**Methods**: All relevant studies utilizing CT-based radiation planning, comparing high-dose (≥ 60 Gy) versus standard-dose (50.4 Gy) radiation for patients with EC were analyzed for this meta-analysis.

**Results**: Eleven studies including 4946 patients met the inclusion criteria, with 96.5% of patients diagnosed with esophageal squamous cell carcinoma (ESCC). The high-dose group demonstrated a significant improvement in local-regional failure (LRF) (OR 2.199, 95% CI 1.487-3.253; P<0.001), two-year local-regional control (LRC) (OR 0.478, 95% CI 0.309-0.740; P=0.001), two-year overall survival (OS) (HR 0.744, 95% CI 0.657-0.843; P<0.001) and five-year OS (HR 0.683, 95% CI 0.561-0.831; P<0.001) rates relative to the standard-dose group. In addition, there was no difference in grade ≥ 3 radiation-related toxicities and treatment-related deaths between the groups.

**Conclusion**: Under the premise of controlling the rate of toxicities, doses of ≥ 60 Gy in CT-based dCCRT of ESCC patients might improve locoregional control and ultimate survival compared to the standard-dose dCCRT. While our review supports a dose-escalation approach in these patients, multiple ongoing randomized trial initial and final reports are awaited to evaluate the effectiveness of this strategy.

## Introduction

Globally, esophageal cancer (EC) is one of the most common causes of cancer-related death 1, 2. Collectively, the 5-year overall survival (OS) rate for all patients is 19% 2. For non-operable, localized EC, definitive concurrent chemoradiotherapy (dCCRT) is usually the standard treatment approach. Currently, the dCCRT radiation dose recommended by National Comprehensive Cancer Network (NCCN) is 50.4 Gy 3, which is primarily based on results of the Radiation Therapy Oncology Group (RTOG) 85-01 and 94-05 randomized trials 4-6. However, the optimal radiation dose in this scenario remains controversial. Many studies have demonstrated that local-regional failure (LRF) remains a common failure pattern for EC patients following dCCRT, most likely to occur within the original gross tumor volume (GTV), even in patients achieving clinical complete response (cCR) following treatment 7-11. In addition, LRF rates of esophageal squamous cell carcinoma (ESCC) appear to be higher than that of esophageal adenocarcinoma (EAC) 12, 13. Because patients with EAC generally have lower cCR rates with chemoradiation and are frequently considered for surgical resection following neoadjuvant treatment (with resultant high rates of local control), these patients have not been the primary focus of dose escalation studies. However, patients with ESCC often have additional comorbidities and generally achieve higher cCR rates with chemoradiation, leading to consideration of non-operative approaches. Therefore, the role of dose escalation is more relevant in patients with ESCC, particularly given that LRF rates are high following standard chemoradiation approaches.

Given the above, radiation dose escalation has been proposed as a technique to obtain higher local-regional control (LRC) and survival rates, notably in Asian countries 14-24. In recent years, multiple investigators have carried out studies comparing the curative impact of high-dose versus standard-dose radiation treatments, although conclusions have been inconsistent 14-31. Additionally, with advances in radiotherapy techniques, questions have been raised as to whether dose escalation utilizing computerized tomography (CT)-based radiotherapy approaches (including 3-Dimensional conformal radiotherapy (3D-CRT), intensity-modulated radiotherapy (IMRT), and proton therapy) could achieve improved outcomes with less toxicity compared to older approaches from the 2-D era. Based on the above, we undertook this meta-analysis to explore whether dose escalation utilizing CT-based radiotherapy techniques could benefit patients with EC receiving dCCRT.

## Methods

Studies published prior to February 2020 comparing radiation dose and disease-related outcomes in nonoperable EC patients were included. Search keywords included “esophageal or oesophageal” and “carcinoma or cancer or neoplasm” and “chemoradiotherapy or chemoradiation or radiochemotherapy or irradiation or chemo-irradiation” and “dose or dose escalation or high dose”. After retrieval, we manually filtered the articles by abstract and/or full text review. Inclusion criteria included: 1) CT-based radiotherapy techniques, such as 3-D, IMRT, or proton beam therapy, were utilized (articles that applied 2D radiotherapy techniques were excluded); 2) dCCRT was used (articles that reported sequential treatments, neoadjuvant or adjuvant CRT combined with surgery, palliative CRT or radiotherapy alone were excluded); 3) subject patients were stratified into high-dose (≥ 60 Gy) and standard-dose (approximately 50 Gy) groups, with comparative data provided; 4) accurate statistical methods, valid data, and clear conclusions were given; 5) hazard ratio (HR) and 95% confidence intervals (CI) were provided or could be calculated.

Outcome data included cCR rate, LRF rate, two-year LRC rate, two- and five-year OS, grade ≥ 3 radiation-related toxicities and treatment-related deaths.

Case-control study evaluation guidelines were applied in order to evaluate the quality of each manuscript for the following criteria: 1) whether gender, age, and tumor location were clearly stated; 2) whether the comparability of the two groups was analyzed; 3) whether the statistical method was appropriate; 4) whether biases were discussed in the study. A point was assigned for each of these four items, with a total score of ≥ 3 indicative of reliable quality. Two researchers independently reviewed the literature according to the unified quality standard, with results crosschecked. If there were some different opinions, a third researcher would be invited to solve the disagreement.

Data were analyzed using Stata version 11.0. Hazard ratio (HR), odds ratio (OR) and 95% CI were used to measure effect size. A Q test was applied to test for result heterogeneity. If P > 0.05, the fixed effect model was used for statistical consolidation. If P ≤ 0.05, the random effect model was used. The combined effect size was tested utilizing the z test. Funnel plots were created to evaluate the risk of publication bias.

## Results

### Literature search and study selection

**Figure 1:**
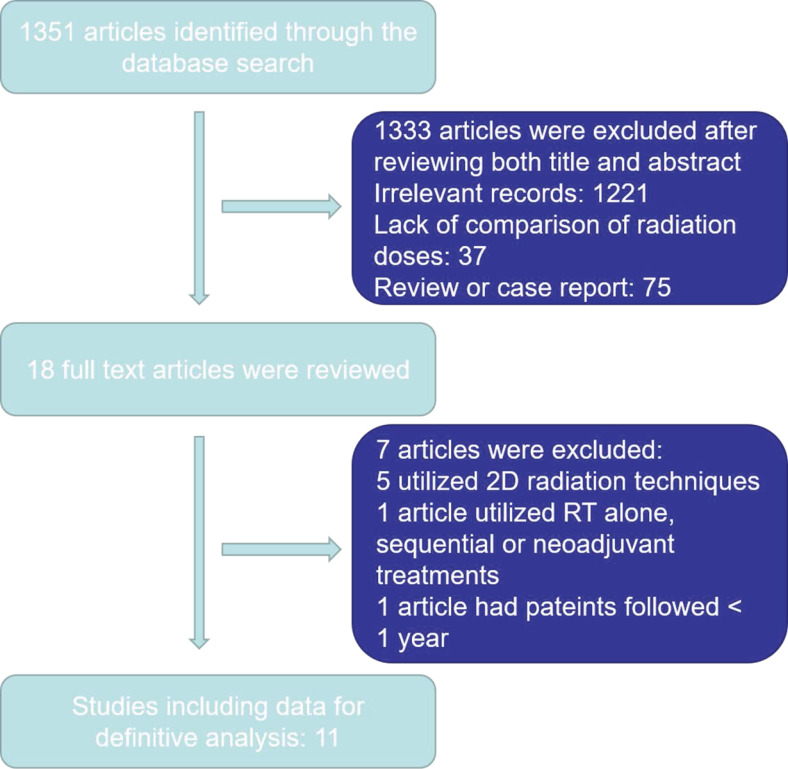
Study selection process.

1351 articles were yielded initially. Ultimately, 1340 articles were excluded as outlined in in *Figure 1*. Eleven articles were selected for the final analysis, including nine retrospective studies and one prospective randomized study 14-19, 23, 25-28* (Table 1).* There were 4946 EC patients included in the analyzed cohort, including 4775 ESCC patients, 142 EAC patients and 29 patients with other histology.

### Outcome data

**Figure 2:**
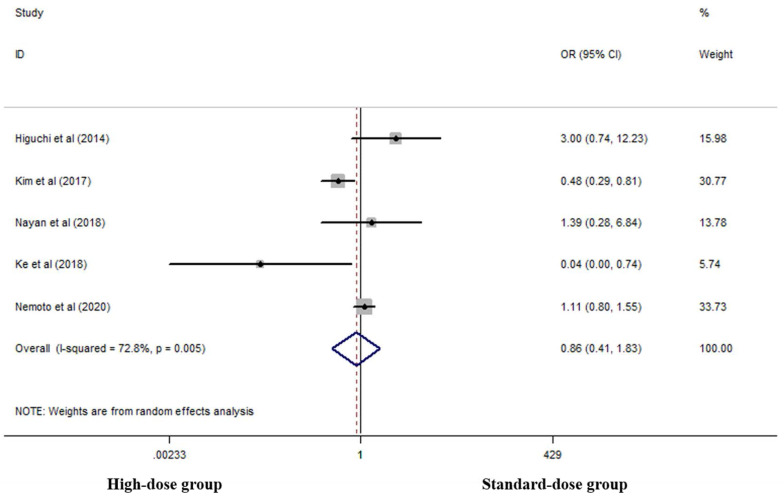
Effects of high- and standard-doses on cCR rate. CI, confidence interval; OR, odds ratio.

Five studies analyzed cCR rates of the two groups 17, 18, 23, 27, 28. There was no difference between the two groups in this respect (OR 0.862, 95% CI 0.406-1.829; P=0.698, *Figure 2*).

**Figure 3:**
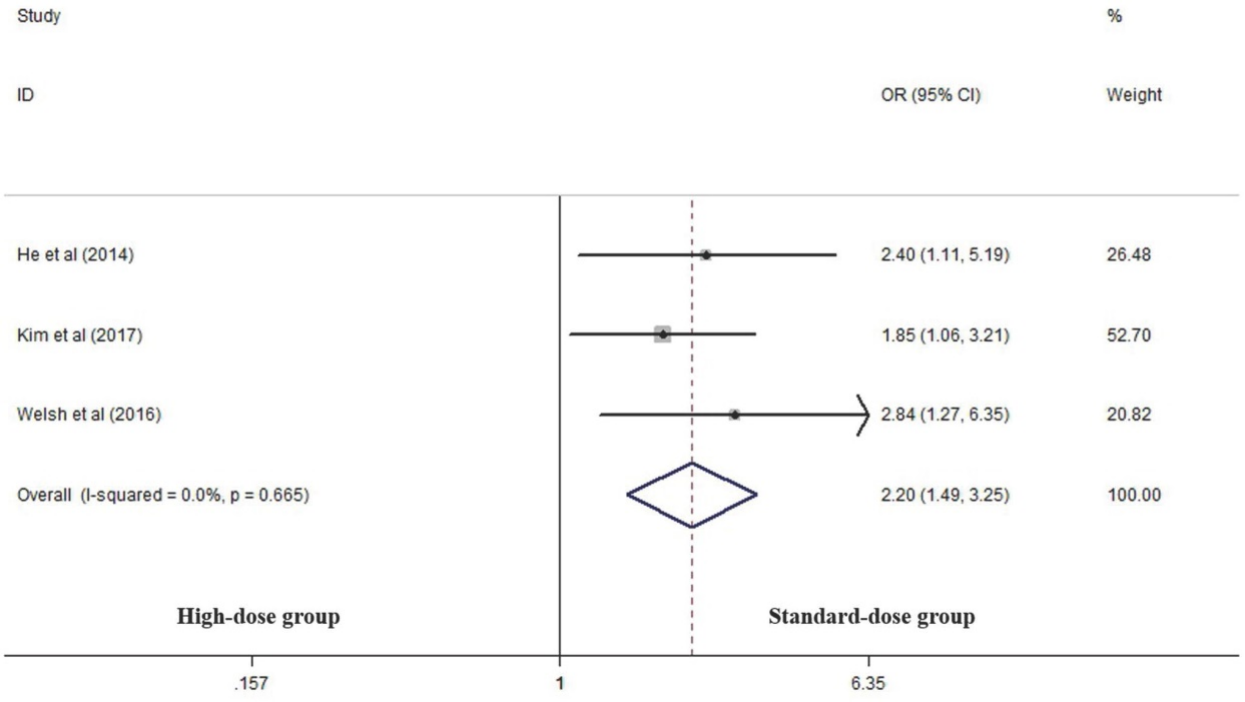
Effects of high- vs. standard-dose RT on local recurrence rate. CI, confidence interval; OR, odds ratio.

Three articles analyzed the LRF rate of the two groups 14, 17, 25. High-dose group had a significant advantage over the standard-dose group in this respect (OR 2.199, 95% CI 1.487-3.253; P<0.001, *Figure 3*).

**Figure 4:**
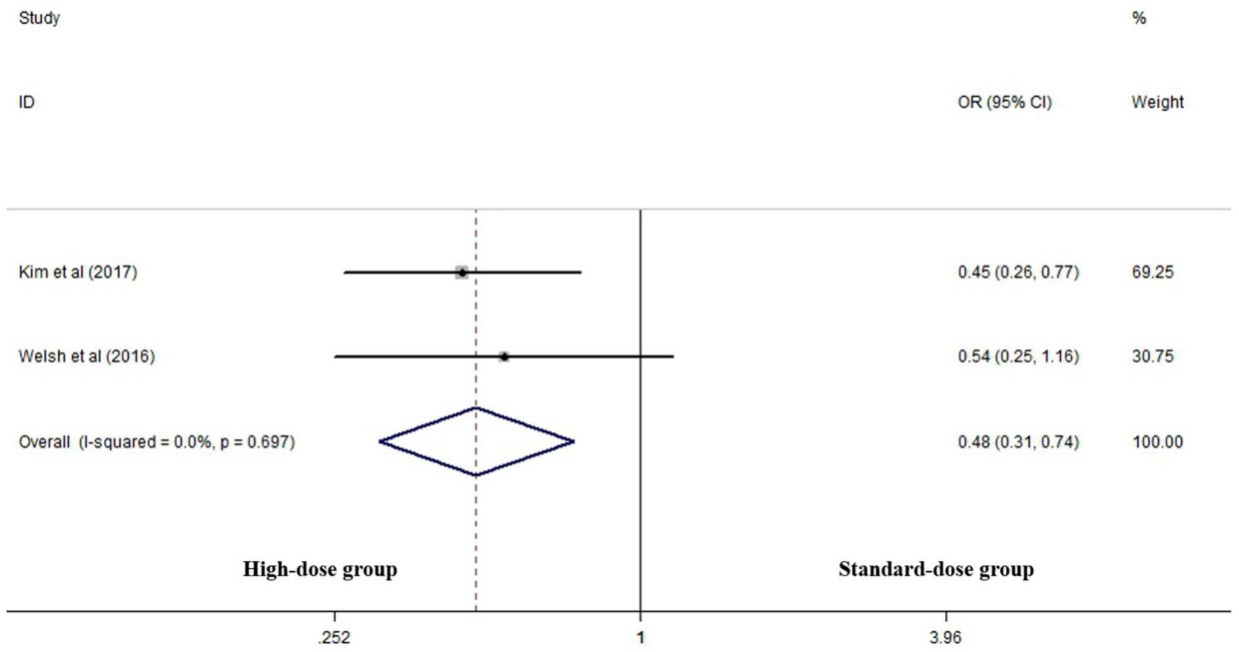
Effects of high- vs. standard-dose RT on two-year local-regional control rate. CI, confidence interval; OR, odds ratio.

Two articles analyzed two-year LRC rates of the two groups 17, 25. High-dose group had a significant advantage over the standard-dose group (OR 0.478, 95% CI 0.309-0.740; P=0.001, *Figure 4*).

**Figure 5:**
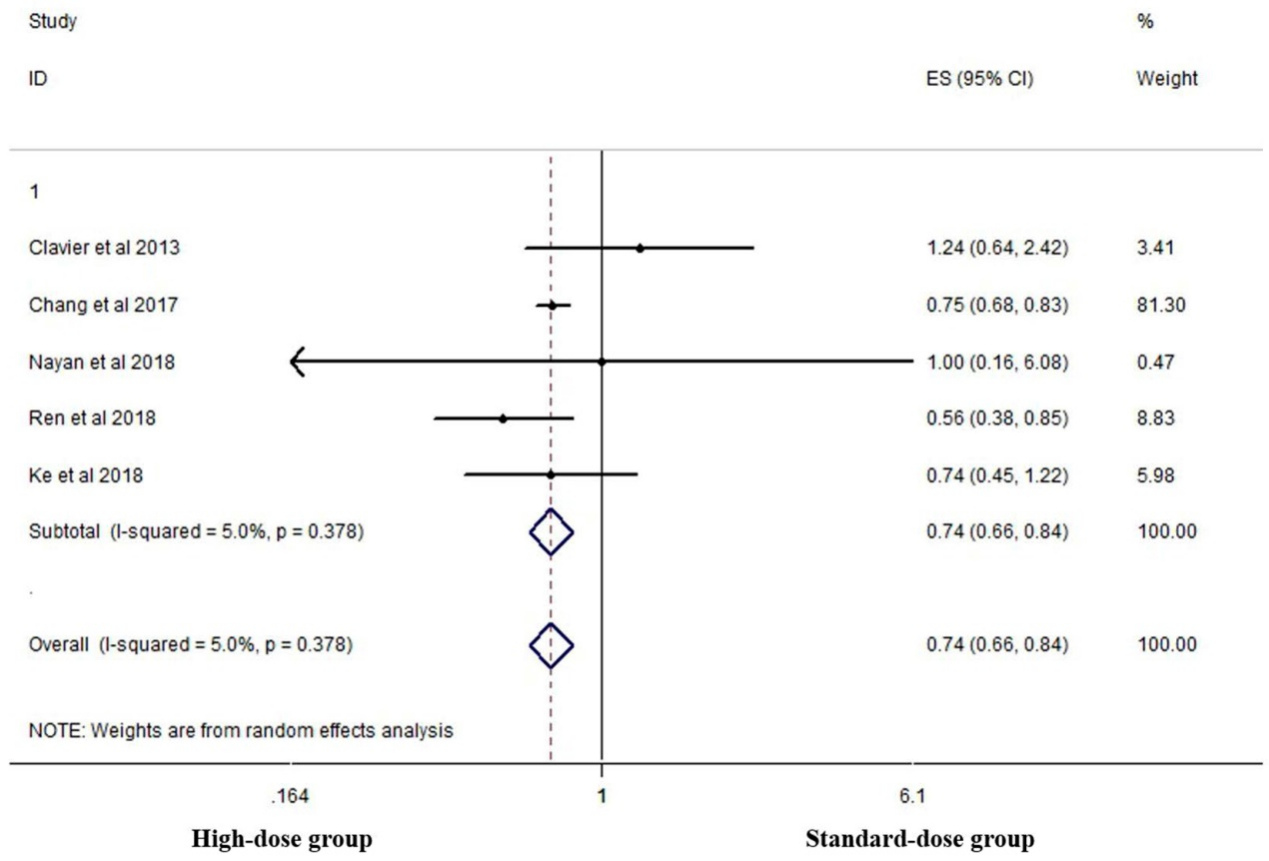
Effects of high- vs. standard-dose RT on two-year OS. CI, confidence interval; ES, effect size.

Five studies analyzed the two-year OS of the two groups 16, 18, 19, 23, 26. High-dose group had a significant advantage over the standard-dose group (HR 0.744, 95% CI 0.657-0.843; P<0.001, *Figure 5*).

**Figure 6:**
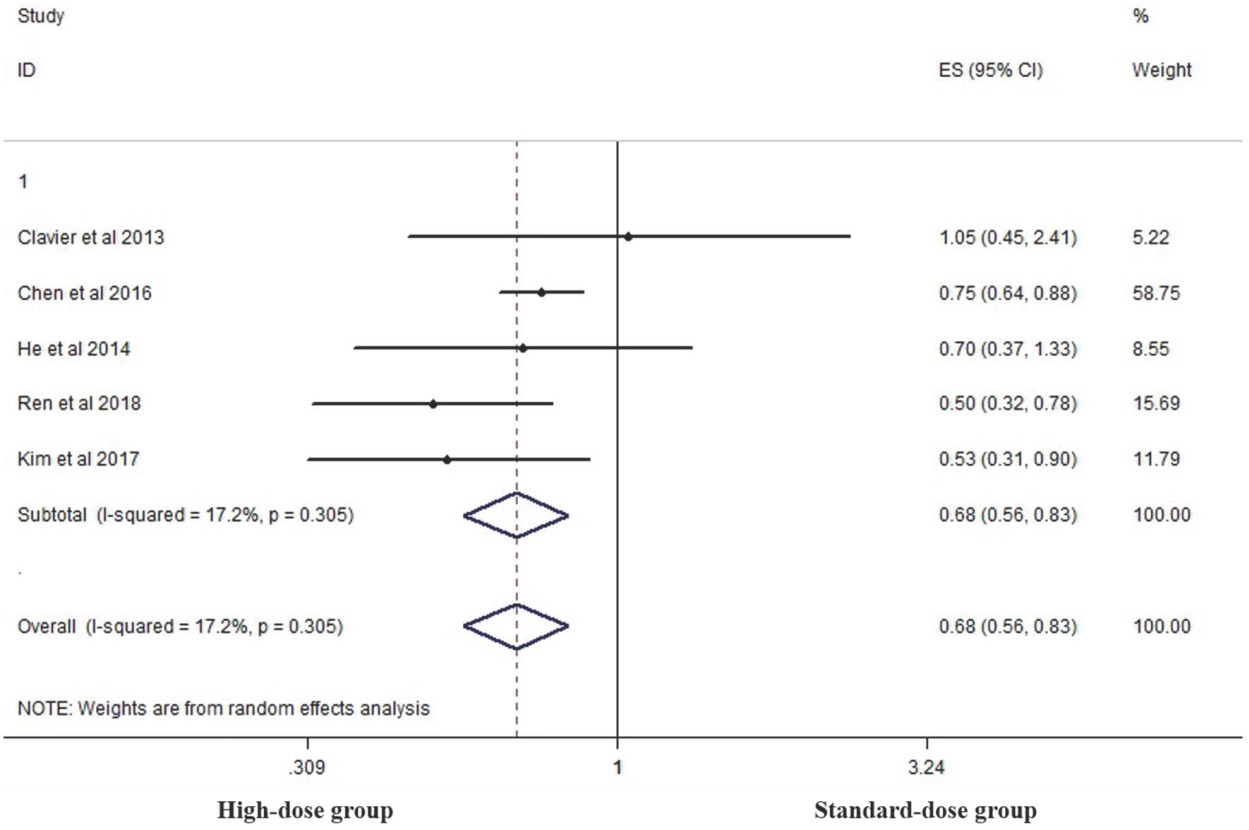
Effects of high- vs. standard-dose RT on five-year OS. CI, confidence interval; ES, effect size.

Five studies analyzed the five-year OS of the two groups 14, 15, 17, 19, 26. High-dose group had a significant advantage over the standard-dose group (HR 0.683, 95% CI 0.561-0.831; P<0.001, *Figure 6*).

Five articles analyzed grade ≥ 3 radiation-related toxicities, with treatment-related deaths evaluated in the four series 14, 17, 19, 23, 27 (*Table 2*). No difference was seen between the two groups in treatment-related deaths (OR 1.026, 95% CI 0.353-2.982; P=0.963), radiation-related esophagitis (OR 0.668, 95% CI 0.385-1.159; P=0.152), pneumonitis (OR 2.654, 95% CI 0.830-8.480; P=0.1), esophageal stenosis (OR 0.578, 95% CI 0.316-1.060; P=0.076) and esophageal fistula (OR 0.927, 95% CI 0.277-3.103; P=0.903).

### Sensitivity analysis

Sensitivity analysis showed that the new combined HR of two-year OS rate were different from the original HR while other items were similar to the original HR/OR (*Table 3*).

### Publication bias analysis

Funnel plot was used to evaluated the publication bias. Egger's regression test was conducted to analyze the symmetry of the funnel plot (*Table 4*). None of the articles demonstrated publication bias (P>0.05).

## Discussion

This study demonstrated that a radiation dose of ≥ 60 Gy utilizing CT-based radiation techniques for the dCCRT of esophageal cancer might decrease LRF and improve two-year LRC, and five-year OS rates of patients without increasing toxicity rates compared to standard RT doses. As there were approximately 97% of patients with ESCC in this study, whether this conclusion was applicable to patients with EAC requires further verification.

RTOG 85-01 and RTOG 94-05/INT 0123 trials established a dose of 50.4 Gy in the dCCRT for patients with inoperable, localized EC 4-6. The RTOG 94-05 trial represents the only large randomized controlled trial (RCT) evaluating RT dose in this setting, comparing the effect of high-dose (64.8 Gy) versus standard-dose (50.4 Gy) treatment. Study results showed that the high-dose arm failed to demonstrate any survival benefit while showing a higher treatment-related mortality rate 6. However, there were caveats, including that seven of the eleven treatment-related deaths in the high-dose group occurred before 50.4 Gy, i.e. dose-escalation was not the cause of death in these patients. Additionally, patients in the high-dose group underwent a treatment break for side effects recovery, resulting in a significantly prolonged treatment time. Finally, patients in the high-dose group received significant lower doses of 5-fluorouracil (5-FU) compared to those in the standard-dose group. These and other factors have the potential to influence ultimate outcomes and potentially negate any benefits of dose-escalation. Additionally, radiation technique in this study was based on what are largely historical 2D approaches, as well as utilized fields larger than that used in contemporary practice, which may increase toxicity rates and not be well suited for dose-escalation.

More recently, improvements in radiotherapy techniques (including CT-based planning), have led to reevaluation of dose-escalation approaches in the dCCRT of esophageal cancer. In a study by Suh et al 21, the high-dose group showed significant improvement in 2-year LRC rate (69% vs. 32%, P<0.01). Ren and colleagues found that the 10-year LRC rate increased by more than 20% (52% vs. 29.8%, P=0.028) with higher radiation doses 19. Similarly, Kim et al demonstrated that their high-dose group experienced significantly lower LRF-alone rates (25.9% vs. 39.2%, P=0.029) and a significantly higher 2-year LRC rate (69.1% vs. 50.3%, p=0.002) relative to the standard-dose group 17. He et al also showed that their high-dose group had a significantly lower LRF rate (17.9% vs. 34.3%, P=0.024) 14. Welsh et al. use simultaneous integrated boost (SIB)-IMRT technique to dose-escalate gross disease and demonstrated that this approach could reduce LRF rates for patients with unresectable locally advanced EC (29% vs. 54%, P=0.01) 25. Along these lines, the current meta-analysis also demonstrated that the high-dose group had significantly lower LRF rates (OR 2.199, 95% CI 1.487-3.253; P<0.001) and higher two-year LRC rates (OR 0.478, 95% CI 0.309-0.740; P=0.001) compared to the standard-dose group.

Given the above, the role of increased LRC on survival benefits has also been explored. Some studies have indicated that higher radiation dose may lead improved survival for ESCC patients undergoing dCCRT 15-17, 19-21, 23-24, while others have not 14, 18, 26-30. A recent registry study reported by Japanese investigators demonstrated 5-year OS rates for stage groups I, II/III and IV were 64.2%/57.2%, 35.0%/27.0% and 18.0%/15.3% in the 50.4 Gy and 60 Gy groups, respectively, with no superiority of the high-dose group in any stage group 28. A meta-analyses by Song et al in 2015 included 55 articles and concluded that high-dose radiation increased treatment response and 5-year OS rates while decreasing LRF and distant failure rates with without additional toxicity, notably for ESCC patients 31. Another study by Chen et al analyzed 18 articles and also found that a dose of ≥ 60 Gy appeared to improve OS and LRC, notably in Asian patients 32. Luo et al. reached a similar conclusion 33. In spite of this, the studies analyzed in these three meta-analyses included many series implementing 2D radiation techniques, which may not reflect the real impact of dose-escalation in modern practice and planning. In the current study, we defined inclusion criteria to include only studies utilizing CT-based radiation planning techniques. Our results demonstrated that a dose of ≥ 60 Gy appeared to increase 5-year OS compared to standard doses, without increasing treatment related toxicities, supporting this strategy in the modern planning approaches in these patients. Based on the current data, the chemotherapy regimens used were not significantly different between the two groups, respectively, in most enrolled studies. While as the limitation of the data, we cannot analyze the effects of the different chemotherapy regimens. As previous researches reported 34, 35, different chemotherapy regimens in the definitive chemoradiotherapy may not affect the survival of patients.

In view of the above results, it is important to review the underlying principles of dose escalation. As reported by Fletcher over four decades ago 36, biologically, doses of 45-50 Gy are generally adequate to control microscopic disease, ≥ 60 Gy required to control gross disease, and nearly 100 Gy to cure solid tumors. In other diseases, including non-small cell lung cancer, it has been estimated that there is an approximate 1% improvement in long-term LRC and 3% decrease in the hazard from death with each additional Gy of radiation delivered 37. To our knowledge, the failure of RTOG 94-05 was related to high rates of toxicities in 2D era. A latest study in Japan used proton beam therapy for esophageal cancer and reported lower rates of toxicities in comparison to photon radiotherapy 38. The biological effective dose (BED) in this study was up to 87.2 Gy (67.2 - 96.1 Gy). The 3-year, 5-year OS rate and five-year LC rate was 66.7%, 56.3% and 64.4%, respectively. The 5-year OS rate based on stage IV were 28.3%. 3D radiotherapy techniques could control the toxicities and side effects, so higher doses may bring survival benefits to patients. In most Asian countries, where ESCC is the predominant histological type, doses of ≥ 60 Gy are much common. Exemplifying this, in the current meta-analysis, ESCC was the predominant histology (approximately 96.5% of patients) and eight of eleven articles included were from Asian countries/only two from the west, making our results particularly relevant to patients with ESCC from Asian countries.

There are limits to this meta-analysis. Firstly, articles were searched among published articles written in English and statistical analysis was limited to the published data. So, publication and language bias maybe exist. Secondly, ten of eleven included studies were retrospective studies (with the inherit limitations), with the one prospective randomized study only included 28 cases. It was a pity that no randomized trial and prospective study with large cases have been published yet (trials ongoing). But two studies with large cases (Chen et al 15 and Ren et al 19) constructed a propensity score matched cohort to balance observable potential confounders. Additional RCTs are needed to verify our conclusion, with many ongoing (*Table 5*). Thirdly, our data include that the combined HR/OR of two-year OS rate were different from the original results following sensitivity analysis. Additionally, we did not perform subgroup analyses with regard to tumor site, stage and pathological type, with locally advanced, thoracic ESCC tumors comprising most of our population. Finally, although cCR is defined as not visible tumor following dCCRT 39, in the clinical practice, specific evaluation methods and criterion of cCR may not be uniform, creating uncertainty as to whether cCR following dCCRT is able to guide subsequent treatment recommendations and requires further study. These factors might have influenced our findings and conclusion.

In conclusion, our study demonstrated that, compared to the standard radiation doses, dose-escalated (≥60 Gy), CT-based radiotherapy techniques might improve ultimate disease-related outcomes in patients with inoperable ESCC under the premise of controlling toxicity rates, and represents the first meta-analysis comparing high- versus standard-radiation doses utilizing CT-based/modern radiotherapy techniques. While our review supports a dose-escalation approach in these patients, multiple ongoing randomized trial initial and final reports are awaited to evaluate the effectiveness of this strategy. In addition, as there were approximately 97% of patients with ESCC in this study, whether this conclusion was applicable to patients with EAC similarly requires further verification.

## Figures and Tables

**Table 1:** Basic characteristics of the included studies

Author	Year	Nation	Study design	SCC/AC	TNM stage	Groups	Patients Number	Radiation dose^#^	Category of RT type
He et al 14	2014	USA	Retrospective	193/0	I-IV	Standard dose	137	Median: 50.4 Gy (41.4-50.4 Gy)	3D-CRT, IMRT and Proton therapy
						High dose	56	Median: 60 Gy (52.2-66 Gy)	
Chen et al 15	2016	China	Retrospective*	648/0	I-IV	Standard dose	324	50-50.4 Gy	3D-CRT and IMRT
						High dose	324	≥60 Gy	
Chang et al 16	2017	China	Retrospective	2061/0	IA-IIIC	Standard dose	1134	Median: 50.4 Gy (45-59.4 Gy)	IMRT
						High dose	927	Median: 66.6 Gy (60-72 Gy)	
Kim et al 17	2017	Korea	Retrospective	230/6	II-III	Standard dose	120	Median: 50.4 Gy (45-59.4 Gy)	3D-CRT and IMRT
						High dose	116	Median: 63 Gy (60-66.6 Gy)	
Nayan et al 18	2018	India	Prospective	28/0	II-III	Standard dose	14	50.4 Gy	3D-CRT and IMRT
						High dose	14	64.8 Gy	
Ren et al 19	2018	China	Retrospective*	380/0	I-IV	Standard dose	190	50.4-54 Gy	3D-CRT and IMRT
						High dose	190	60 Gy	
Ke et al 23	2018	China	Retrospective	84/0	I-III	Standard dose	42	Median: 49.5 Gy (44-50.4 Gy)	IMRT and Conformal Arc
						High dose	42	Median: 61.8 Gy (52.2-70 Gy)	
Welsh et al 25	2016	USA	Retrospective	45/89	I-IV	Standard dose	97	50.4 Gy	IMRT and Proton therapy
						High dose	38	Median: 63 Gy (58.8-63 Gy)	
Clavier et al 26	2013	France	Retrospective	113/30	I-IV	Standard dose	60	Median: 50Gy (38-50.4 Gy)	3D-CRT and IMRT
						High dose	83	Median: 66Gy (50.7-72 Gy)	
Higuchi et al 27	2014	Japan	Retrospective	42/0	I-IV	Standard dose	30	50.4 Gy	3D-CRT and IMRT
						High dose	12	61.2 Gy	
Nemoto et al 28	2020	Japan	Retrospective	951/17	I-IV	Standard dose	171	50.4Gy	3D-CRT and IMRT
						High dose	825	60Gy	

SCC, squamous cell carcinoma; AC, adenocarcinoma; 3D-CRT, 3-Dimensional conformal radiotherapy; IMRT, Intensity-modulated radiation therapy; # Median radiation dose and the dose range of the whole group. *Construct a propensity score matched cohort (1:1 for high dose vs standard dose).

**Table 2:** Grade ≥ 3 radiation-related toxicities and treatment-related deaths

Article	Treatment-related death^#^	Esophagitis^#^	Pneumonitis^#^	Esophageal stenosis^#^	Esophageal fistula^#^
He et al 14	5.1%/3.6%	20.4%/17.9%	6.6%/0%	18.3%/32.1%	2.2%/3.6%
Kim et al 17	1.7%/0.9%	--	1.7%/0%	5%/5.2%	1.7%/1.7%
Ren et al 19	0.5%/1.6%	2.6%/7.4%	2.1%/2.6%	--	--
Higuchi et al 27	--	23.3%/41.6%	--	--	6.7%/0%
Ke et al 23	0%/0%	0%/0%	0%/0%	0%/0%	0%/0%

# standard-dose group/high-dose group.

**Table 3:** Sensitivity analysis

Item	Deleted article	HR/OR	95% CI	*P*
cCR rate	Nemoto et al 28	0.734	0.206 - 2.617	0.633
LRF rate	Kim et al 17	2.593	1.485 - 4.530	0.001
Two-year LRC rate	--	--	--	--
Two-year OS	Chang et al 16	0.748	0.523 - 1.968	0.11
Five-year OS	Chen et al 15	0.591	0.444 - 0.787	<0.001
Treatment-related death	Ren et al 19	1.588	0.416 - 6.062	0.499
Esophagitis	Ren et al 19	0.935	0.471 - 1.856	0.847
Pneumonitis	Ren et al 19	6.874	0.847 - 55.762	0.071
Esophageal stenosis	--	--	--	--
Esophageal fistula	He et al 14	1.266	0.250 - 6.414	0.776

HR, hazard ratio; OR, odds ratio; CI, confidence interval; cCR clinical complete response; LRF, local-regional failure; OS, overall survival.

**Table 4:** Publication bias results of selected articles

Evaluation Items	t	95% CI	*P*
cCR rate	0.25	-8.056 - 9.037	0.828
LRF rate	4.53	-5.327 - 11.238	0.138
Two-year LRC rate	--	--	--
Two-year OS	0.28	-2.113 - 2.520	0.798
Five-year OS	-0.68	-3.529 - 2.290	0.546
Treatment-related death	-0.37	-45.214 - 42.684	0.777
Esophagitis	-1.14	-46.51 - 38.834	0.458
Pneumonitis	3.69	-6.556 - 11.931	0.168
Esophageal stenosis	--	--	--
Esophageal fistula	2.84	-6.389 - 10.059	0.216

CI, confidence interval; cCR, clinical complete response; LRF, local-regional failure; OS, overall survival.

**Table 5:** Selected ongoing RCTs evaluating dose escalation

NCT number	Country	Start Date	Estimated Completion Date	Groups	Estimated Enrollment
NCT 01348217	France	2011	2019	66 Gy vs. 50 Gy	252
NCT 01937208	China	2013	2017	60 Gy vs. 50 Gy	300
NCT 02556762	China	2015	2021	66/50 Gy (SIB) vs. 50 Gy	202
NCT 02741856	UK	2016	2023	60 Gy vs. 50 Gy	584
NCT 02850991	China	2016	2021	59.4 Gy vs. 50.4 Gy	308
NCT 03790553	China	2018	2025	61.2 Gy vs. 50.4 Gy	646
